# The relative importance of friendship to happiness increases with age

**DOI:** 10.1371/journal.pone.0288095

**Published:** 2023-07-13

**Authors:** Inmyung Song, Jin-Won Kwon, Soo Min Jeon

**Affiliations:** 1 Department of Health Administration, Kongju National University, Gongju, South Korea; 2 BK21 Four Community-Based Intelligent Novel Drug Discovery Education Unit, College of Pharmacy and Research Institute of Pharmaceutical Sciences, Kyungpook National University, Daegu, South Korea; 3 Jeju Research Institute of Pharmaceutical Sciences, College of Pharmacy, Jeju National University, Jeju, South Korea; Centre for Demographic Studies, SPAIN

## Abstract

Happiness is predicted by social relationships in general and contact frequency in particular. This study aims to examine if the relative importance of social contacts with the closest family/relative, friend, and neighbor in happiness changes with advancing age. We used data for all participants aged 19 years and older (n = 229,099) in the 2019 Community Health Survey, which measured the frequency of contact with the closest relative/family, neighbor, and friend among a representative sample of Koreans between August 16 and October 31, 2019. The Shapley value decomposition method was used to measure the relative importance of each predictor of happiness. Overall, contact frequency was positively associated with happiness (*p*<0.001). The relative importance value of contact with the closest family, neighbor, and friend to happiness increased from 4.70%, 3.98%, and 7.35%, respectively, in the 19–29 years group to 8.09%, 4.44%, and 11.00%, respectively, in the 60 years and older group. Frequent interactions with the closest friend could have a greater impact on happiness in old age than those with the closest family and neighbor.

## Introduction

There is growing evidence that social relationships are associated with happiness. How much one enjoys and derives satisfaction from close social relationships is a significant predictor of subjective well-being and life satisfaction [1]. People who value social relationships are more likely to be happy than those who value achievements in other life domains, such as education and career [2]. The strength of family and relationships with friends is related to happiness and life satisfaction, directly as well as indirectly through an impact on health [3]. Feeling connected socially has a positive influence not only on psychological well-being but also on physical well-being [4]. The positive impact of increasing interactions with friends and relatives on life satisfaction is so great that an effort was made to estimate a monetary value [5].

Researchers have long tried to explore why social relationships are important to happiness [6]. One hypothesis is that individuals have a strong need to belong, which may be satisfied by building stable and strong interpersonal relationships [7]. The quality of friendship plays an important role in happiness because quality friendship satisfies psychological needs to belong and feel capable [8]. People desire to have close friends who are understanding and whom to talk to in times of needs, and this particular aspect of social connectedness was dubbed relational connectedness by Hawkley et al. (2005) [9]. In addition to satisfying the fundamental need to belong, social connectedness can provide social support, which is composed of emotional support (e.g., empathy) and instrumental support [10]. The latter refers to tangible assistance provided by friends and family to meet the needs of individuals for help. The two dimensions of social support are shown to interact with each other such that the provider of practical support gets psychological well-being benefit only when he/she is engaged emotionally [11]. There is empirical evidence that the sense of belonging, giving, and reciprocating help all were associated with happiness among Korean adults [12].

Researchers have examined social connections as a predictor of happiness by using a variety of measures, such as the size of social network, the number of friends, the frequency of social activities [6], membership in various kinds of groups and associations, marital status, trust in individuals, and social contact [13]. Social contact, in particular, was defined in terms of number of friends, closeness of network, and contact frequency [14]. Contact frequency was not as important in predicting happiness and life satisfaction as the quality of relationship, such as how closely one feels connected with friends and family, in older African Americans [15] and Canadian adults [16]. Nonetheless, there is evidence that the intensity of friendship, as measured by contact frequency, plays an important role in life satisfaction [17]. According to an analysis of data from 22 countries, the frequency of contact with family, relatives, and friends was a strong predictor of happiness [18].

It appears that the different types of social contact have a varying degree of impact on one’s subjective well-being. For example, relative well-being benefits are greatest in the order of frequent interactions with friends, family, and neighbors in Canada and the United States [3]. Among social relationships, friendships received a particular attention from researchers as friends could play an important role in one’s happiness for a variety of reasons. One can do activities together with friends, which can contribute to happiness [19]. As a voluntary interdependent relationship, friendship can also promote companionship, intimacy, affection, and mutual assistance [20]. Some researchers focused on not just any friendship but best friendship. Among college students in the United States, happiness is predicted not by the number of friends but by the quality of best friendship, which represents offering help and fostering intimacy and companionship [19]. Among all features of best friendship examined, companionship was the strongest predictor of happiness [21].

The impact of social contact on happiness may vary depending on the life circumstances of individuals. This premise has been explored in the aged population and people living alone. Older Americans with a network of a large number of friends were more likely to be happy than their peers with a restricted social network [22]. Family contact was positively associated with life satisfaction in older African Americans [15]. Having active interactions with friends and family was shown to have a positive impact on subjective well-being of the elderly [23]. The frequency of contact with friends increased the odds of feeling happy among Japanese living alone [24]. The frequency of contact with family was more important for happiness among older Koreans living alone, whereas the frequency of contact with friends increased the odds of feeling happy among older Koreans living with their spouse [25].

As people age, they experience the loss of roles that they have previously played in the workplace and at home. By compensating for the associated sense of loss, friendships can be beneficial to the psychological well-being of the elderly [26]. However, the benefits of friendships in older age extend beyond psychological well-being and include cognitive and physical functioning that social interactions can boost [27]. In addition to role losses, old people confront many health problems, which may require assistance and caregiving from others. Therefore, actively connecting with friends and neighbors can contribute to the sense of independence for older adults [28].

Researchers explored the source of social support and its differential mechanism for enhancing the emotional well-being of the elderly. For example, spouse support is more important in decreasing negative affect, whereas friendship has a greater influence on increasing positive affect [29]. Friends have a greater impact on the morale of the elderly than their grandchildren [30]. Similarly, the quality of relationships with friends was more important for life satisfaction of older adults than that with their children [16].

In this context, this study was designed to investigate the predictors of happiness, with a focus on the frequency of contact with the closest family, neighbor, and friend. The research question of particular interest is whether the relative importance of the type of social contact changes across age groups. Having reviewed the literature on happiness as related to social relationships, we developed the following hypotheses.

1. The frequency of social contact with the closest family/relative, friend, and neighbor is positively associated with happiness.
2. The relative importance of the type of social contact to happiness changes across age groups.

## Methods

### Data

This secondary data analysis relied on data on contact frequency that were collected by the Korea Centers for Disease Control and Prevention in the Community Health Survey (CHS). The annual survey that began in 2008 collects data using in-person interviews. The CHS is administered to a representative sample of Korean adults aged 19 years and older. The 2019 CHS measured social relationships in one dimension of contact frequency, that is, the frequency of meeting or contacting with the closest relative (including the family member), neighbor, and friend. Contact frequency was measured by the question “How often do you see or contact with the closest relative/family, neighbor, and friend that you have contacted most often?” Response choices comprise ‘less than once per month’, ‘once per month’, ‘two or three times per month’, ‘once a week’, ‘two or three times a week’, and ‘four or more times per week’.

The CHS uses a complex survey design in which administrative areas were cluster-sampled using residential type as a stratification variable. A sample of households within each area were selected using systematic sampling. All members aged 19 and older of selected households were interviewed. All analyses were adjusted for sampling weights. The 2019 CHS surveyed a total of 229,099 adults aged 19 years and older between August 16 and October 31, 2019 [31]. All participants in the 2019 CHS were used for analysis.

### Happiness measure

Happiness, which is often used interchangeably with subjective well-being, has been defined as consisting of three dimensions: global life satisfaction, presence of positive affect and absence of negative affect [32]. However, in the 2019 CHS, happiness was measured in one item question of “all things considered, how happy are you in life these days?” The respondent was asked to rate happiness on a scale of one to ten, where ten represents the happiest state and one the unhappiest state. We used the single item happiness score as the dependent variable in regression models.

### Control variables

Previously, happiness is shown to be influenced by socio-demographic variables like sex, marital status, education level, living arrangement, employment status, and household income [33, 34]. This present study examined the impact of contact frequency on happiness while controlling for the socio-demographic factors. Marital status was grouped into currently married, formerly married, and never-married. The formerly married group comprises divorced, widowed, and separated. Education level was grouped into up to elementary, middle, high school, and college and higher education. Living arrangement was dichotomized into living alone and cohabitating with others. Employment status was grouped into salaried, self-employed, and economically inactive. Household income (in KRW 10,000) was log-transformed to account for the skewness of income data distribution. In addition to socio-economic variables, this study also controlled for subjective health status, which was measured on a five-point Likert scale (very healthy, healthy, neutral, unhealthy, and very unhealthy) using a single question in the 2019 CHS, “how would you assess your health in general?”

### Statistical analysis

We first described the study population according to sociodemographic characteristics, subjective health status, and social contact variables. Then, we compared the mean happiness levels across the groups of different characteristics. We also estimated several ordinary least square (OLS) regression models for all ages as well as by age group. OLS has been implemented to estimate predictors of happiness and life satisfaction in the existing studies [34, 35]. Happiness measured in the Cantril ladder scale has been treated as a theoretically equal interval and continuous measure of life satisfaction [35], justifying the use of ordinary least square models. The Cantril ladder scale employs a visual ladder-like tool with equal intervals drawn on the questionnaire. A similar visual tool was used to measure happiness in the Community Health Survey. One could argue that the ideal method would be to treat happiness as an ordinal scale variable, which would require implementing ordered probit models. However, the models produce pseudo R^2^, which was difficult to decompose by using the methods described below.

OLS produces R^2^, a measure of goodness-of-fit, which represents the total amount of variance in the dependent variable explained by all independent variables included in the model. The dependent variable in all regression models was a measure of happiness. The regression model for all ages was run with independent variables including sex, age, education level, marital status, living alone (yes or no), employment status (self-employed, salaried, economically inactive), log household income, subjective health status, number of contacts with the closest relative/family, the neighbor, and the friend. To test if age moderates the effect of contact frequency on happiness, we implemented a model with the interaction term, age*contact frequency. Regression models by age group omitted age as an independent variable.

To determine the relative importance of the predictors of happiness, we calculated the Shapley value by adopting the empirical strategy used by Lamu and Olsen (2016). While a common approach is to interpret coefficients as values representing the importance of predictors in a regression model, research on the complex concept of happiness is susceptible to correlations among predictors [1]. Therefore, we used the Shapley value method to decompose variations in regression models, which is a technique that has game theory at the foundation [36].

The Shapley value evaluates the marginal contribution of each predictor to happiness. The value is calculated for each predictor by comparing R^2^ with or without the predictor in every possible OLS model. The sum of Shapley values for all predictors in a model is equal to the R^2^ of the model. %R^2^ measures the proportional contribution of each predictor to R^2^ and sums to 100%. As a share of goodness-of-fit, %R^2^ represents the relative importance value of each predictor of happiness. To determine how the marginal contribution of each predictor to happiness changes with advancing age, we divided respondents into four age groups: 19–29, 30–39, 40–59, and 60 years and older. Young adults under 30 years were categorized as a group as they may share socioeconomic characteristics in terms of schooling, employment, and marital status that are different from other age groups. A regression model was run for each age group. Shapley value and %R^2^ with 95% confidence interval were computed using 1,000 bootstrap samples for each age group.

All statistical analyses were performed by using SAS version 9.4 (Cary, NC, USA) and R software 3.6.1 (R foundation, Vienna, Austria). The Institutional Review Board of Kongju National University approved the study protocol and waived the requirement for informed consent (reference No. KNU_IRB_2022–074).

## Results

Of the study population, 50.4% were female (Table 1). 26.8% were aged 60 years and older. 42.0% attained college or higher education and 63.9% were currently married. 11.2% lived alone and 47.0% were salaried workers. 15.0% rated their general health to be either very unhealthy or healthy. 18.1%, 47.4%, and 16.7% of Korean adults did not have any contact per month with the closest relative/family, neighbor, and friend, respectively.

**Table 1:** Characteristics of study subjects (n = 229,099).

Variable	Category	N	% (weighted)
Sex	Male	102,572	49.6
	Female	126,527	50.4
Age group, years	19–29	23,383	17.3
	30–39	26,712	16.4
	40–59	80,082	39.5
	≥60	98,922	26.8
Education level	No or primary education	57,901	12.7
	Middle school	25,505	8.2
	High school	75,409	37.1
	College or higher	70,060	42.0
Marital status	Currently married	152,095	63.9
	Ever-married	41,416	12.4
	Never-married	35,372	23.7
Live alone	No	194,090	88.8
	Yes	34,835	11.2
Employment status	Self-employed	43,153	14.8
	Salaried	85,855	47.0
	Economically inactive	94,420	38.2
Subjective health status	Very healthy	10,590	5.5
	Healthy	67,216	33.2
	Neutral	102,443	46.2
	Unhealthy	39,229	12.5
	Very unhealthy	9,604	2.5
Contact frequency with relative/family	<1/month	34,748	18.1
	1/month	27,685	14.5
	2–3/month	32,099	15.0
	1/week	28,911	12.6
	2–3/week	38,537	14.7
	≥4/week	67,039	25.2
Contact frequency with neighbor	<1/month	73,594	47.4
	1/month	14,428	7.6
	2–3/month	14,692	7.0
	1/week	17,570	7.6
	2–3/week	32,158	11.6
	≥4/week	75,357	18.8
Contact frequency with friend	<1/month	45,724	16.7
	1/month	28,886	13.3
	2–3/month	30,814	14.8
	1/week	24,592	11.9
	2–3/week	40,378	18.4
	≥4/week	58,465	25.0

The frequency (N) is based on the sample. % is estimated for the population by adjusting for the sampling weights in the complex survey data.

On average, men were happier than women (*p*<0.001, Table 2). The mean happiness level peaked in 30–39 years and increased with higher education, better subjective health, and more frequent contacts with the closest relative/family and the friend (*p*<0.001). The currently married and individuals that were cohabitating with others were happier than their counterparts.

**Table 2:** Mean happiness score by sociodemographic characteristics, subjective health, and contact frequency.

Variable	Category	Mean	SE	*p*-value	95% CI
Sex	Male	7.04	0.01	< .001	0.05–0.09*
	Female	6.97	0.01		NA
Age group, years	19–29	7.02	0.01	< .001	0.23–0.30*
	30–39	7.21	0.01		0.43–0.49*
	40–59	7.08	0.01		0.30–0.35*
	≥60	6.75	0.01		NA
Education level	No or primary education	6.45	0.01	< .001	NA
	Middle school	6.67	0.02		0.18–0.26*
	High school	6.93	0.01		0.45–0.50*
	College or higher	7.30	0.01		0.81–0.87*
Marital status	Currently married	7.18	0.01	< .001	0.32–0.37*
	Ever-married	6.40	0.01		-0.47 –-0.40*
	Never-married	6.84	0.01		NA
Live alone	No	7.06	0.01	< .001	0.53–0.60*
	Yes	6.50	0.01		NA
Employment status	Self-employed	7.09	0.01	< .001	0.24–0.30*
	Salaried	7.12	0.01		0.28–0.32*
	Economically inactive	6.82	0.01		NA
Subjective health status	Very healthy	7.92	0.02	< .001	2.59–2.76*
	Healthy	7.48	0.01		2.16–2.31*
	Neutral	6.87	0.01		1.56–1.70*
	Unhealthy	6.16	0.01		0.84–0.99*
	Very unhealthy	5.24	0.03		NA
Contact frequency with relative/family	<1/month	6.59	0.01	< .001	NA
	1/month	6.96	0.01		0.33–0.41*
	2–3/month	7.06	0.01		0.44–0.51*
	1/week	7.09	0.01		0.47–0.55*
	2–3/week	7.11	0.01		0.48–0.55*
	≥4/week	7.19	0.01		0.57–0.64*
Contact frequency with neighbor	<1/month	6.88	0.01	< .001	NA
	1/month	7.09	0.02		0.17–0.25*
	2–3/month	7.09	0.02		0.18–0.25*
	1/week	7.12	0.02		0.20–0.28*
	2–3/week	7.12	0.01		0.21–0.27*
	≥4/week	7.12	0.01		0.21–0.27*
Contact frequency with friend	<1/month	6.53	0.01	< .001	NA
	1/month	6.94	0.01		0.37–0.45*
	2–3/month	7.06	0.01		0.49–0.56*
	1/week	7.10	0.01		0.53–0.60*
	2–3/week	7.12	0.01		0.55–0.62*
	≥4/week	7.18	0.01		0.61–0.68*

Abbreviations: CI, confidence intervals; NA, not available

*Post-hoc analysis was significant at the 0.001 level.

In the models for all ages and the ≥ 60 years group, having more frequent contacts with the closest relative/family was consistently and positively associated with happiness (Tables 3 and 4, *p*<0.001). The regression model with interaction terms showed that age and contact frequency had a positive interaction effect on happiness. Models for the younger groups (19–29, 30–39, and 40–59 years) show that people do not become happier by having more contacts with the closest relative/family (Table 4). However, having more contacts with the closest friend consistently increased happiness in all age groups. The relative importance value of contacting with the closest family, neighbor, and friend to happiness increased from 4.70%, 3.98%, and 7.35%, respectively, in the 19–29 years group to 8.09%, 4.44%, and 11.00%, respectively, in the 60 years and older group (Table 5).

**Table 3:** Regression models with or without interaction terms.

		Model without interaction terms			Model with interaction terms		
Variable (reference)	Category	B		SE	B		SE
Sex (female)	Male	0.00		0.01	0.00	***	0.01
Age	(years)	0.00		0.00	-0.01	*	0.00
Education level	Middle school	-0.06	^***^	0.02	-0.04	**	0.02
(no or primary education)	High school	0.11	^***^	0.01	0.14	***	0.01
	College or higher	0.38	^***^	0.01	0.42	***	0.01
Marital status	Currently married	0.46	^***^	0.01	0.51	***	0.01
(never-married)	Ever-married	0.11	^***^	0.02	0.15	***	0.02
Live alone (yes)	No	0.14	^***^	0.01	0.13	***	0.01
Employment status	Self-employed	-0.05	^***^	0.01	-0.04	***	0.01
(economically inactive)	Salaried	0.03	^**^	0.01	0.03	***	0.01
Log household income	(10,000 won)	-0.02	^***^	0.00	-0.02	***	0.00
Subjective health status	Very healthy	2.43	^***^	0.03	2.43	***	0.03
(very unhealthy)	Healthy	1.96	^***^	0.02	1.95	***	0.02
	Neutral	1.37	^***^	0.02	1.36	***	0.02
	Unhealthy	0.77	^***^	0.02	0.77	***	0.02
Contact frequency with relative/family (<1/month)	1/month	0.20	^***^	0.01	0.14	***	0.03
	2–3/month	0.28	^***^	0.01	0.21	***	0.03
	1/week	0.30	^***^	0.01	0.11	***	0.04
	2–3/week	0.32	^***^	0.01	0.07	**	0.04
	≥4/week	0.38	^***^	0.01	0.05	*	0.03
Contact frequency with neighbor	1/month	0.13	^***^	0.01	0.10	***	0.04
(<1/month)	2–3/month	0.11	^***^	0.01	0.05	***	0.04
	1/week	0.14	^***^	0.01	0.11	**	0.04
	2–3/week	0.18	^***^	0.01	0.07	**	0.04
	≥4/week	0.29	***	0.01	-0.06	*	0.03
Contact frequency with friend	1/month	0.15	***	0.01	0.09	*	0.04
(<1/month)	2–3/month	0.21	***	0.01	0.18	***	0.04
	1/week	0.26	***	0.01	0.21	***	0.04
	2–3/week	0.30	***	0.01	0.34	***	0.04
	≥4/week	0.41	***	0.01	0.54	***	0.03
Age*contact frequency with relative/family (<1/month)	1/month				0.00	*	0.00
	2–3/month				0.00	*	0.00
	1/week				0.00	***	0.00
	2–3/week				0.01	***	0.00
	≥4/week				0.01	***	0.00
Age*contact frequency with neighbor (<1/month)	1/month				0.00		0.00
	2–3/month				0.00	*	0.00
	1/week				0.00		0.00
	2–3/week				0.00	***	0.00
	≥4/week				0.01	***	0.00
Age*contact frequency with friend (<1/month)	1/month				0.00		0.00
	2–3/month				0.00		0.00
	1/week				0.00		0.00
	2–3/week				0.00		0.00
	≥4/week				0.00	***	0.00
Constant		4.46			4.61		
Observations		221,187			22,187		
R^2^		0.154			0.155		

* *p*<0.05

** *p*<0.01

*** *p*<0.001

**Table 4:** Unstandardized regression coefficients for happiness.

		19–29 years			30–39 years			40–59 years			≥60 years		
Variable (reference)	Category	B		SE	B		SE	B		SE	B		SE
Sex (female)	Male	0.19	***	0.02	0.14	***	0.02	-0.13	***	0.01	-0.13	***	0.01
Education level	Middle school	-1.23	***	0.31	-0.26		0.21	0.02		0.04	0.06	***	0.02
(no or primary education)	High school	-0.55		0.30	-0.15		0.19	0.22	***	0.03	0.16	***	0.01
	College or higher	-0.51		0.30	0.17		0.19	0.56	***	0.03	0.61	***	0.02
Marital status	Currently married	0.52	***	0.04	0.64	***	0.02	0.66	***	0.02	0.61	***	0.06
(never-married)	Ever-married	-1.09	***	0.19	-0.03		0.06	0.03		0.03	0.47	***	0.06
Live alone (yes)	No	0.05		0.03	0.14	***	0.04	0.15	***	0.02	0.18	***	0.02
Employment status	Self-employed	0.04		0.06	0.12	***	0.03	0.11	***	0.02	0.00		0.02
(economically inactive)	Salaried	0.04		0.02	0.20	***	0.02	0.19	***	0.01	-0.05	***	0.01
Log household income	(10,000 won)	-0.03	**	0.01	-0.03	***	0.01	-0.02	***	0.00	-0.01	*	0.01
Subjective health status	Very healthy	3.01	***	0.18	2.86	***	0.15	2.39	***	0.06	2.35	***	0.04
(very unhealthy)	Healthy	2.52	***	0.18	2.47	***	0.15	2.00	***	0.05	1.88	***	0.02
	Neutral	1.81	***	0.18	1.92	***	0.15	1.49	***	0.05	1.28	***	0.02
	Unhealthy	1.10	***	0.18	1.28	***	0.15	0.91	***	0.05	0.70	***	0.02
Contact frequency with relative/family	1/month	0.19	***	0.03	0.17	***	0.03	0.16	***	0.02	0.28	***	0.02
(<1/month)	2–3/month	0.26	***	0.03	0.26	***	0.03	0.25	***	0.02	0.33	***	0.02
	1/week	0.20	***	0.04	0.24	***	0.03	0.28	***	0.02	0.39	***	0.02
	2–3/week	0.21	***	0.04	0.27	***	0.03	0.28	***	0.02	0.44	***	0.02
	≥4/week	0.25	***	0.03	0.33	***	0.03	0.34	***	0.02	0.53	***	0.02
Contact frequency with neighbor	1/month	0.17	***	0.04	0.09	*	0.04	0.12	***	0.02	0.15	***	0.02
(<1/month)	2–3/month	0.18	***	0.05	0.04		0.04	0.12	***	0.02	0.14	***	0.02
	1/week	0.17	**	0.05	0.15	***	0.03	0.13	***	0.02	0.17	***	0.02
	2–3/week	0.26	***	0.05	0.15	***	0.03	0.19	***	0.02	0.20	***	0.02
	≥4/week	0.24	***	0.04	0.25	***	0.03	0.28	***	0.02	0.35	***	0.01
Contact frequency with friend	1/month	0.17	***	0.06	0.10	***	0.03	0.12	***	0.02	0.21	***	0.02
(<1/month)	2–3/month	0.27	***	0.05	0.15	***	0.03	0.17	***	0.02	0.28	***	0.02
	1/week	0.32	***	0.05	0.20	***	0.03	0.23	***	0.02	0.32	***	0.02
	2–3/week	0.41	***	0.05	0.21	***	0.03	0.24	***	0.02	0.35	***	0.02
	≥4/week	0.55	***	0.05	0.28	***	0.03	0.28	***	0.02	0.45	***	0.02
Constant		4.78			4.06			4.03			4.10		
Observations		22,390			25,795			77,397			95,605		
R^2^		0.145			0.150			0.154			0.167		

**Table 5:** Relative importance of the predictors of happiness by age group.

	19–29 years			30–39 years				40–59 years				≥ 60 years			
Variable	SV	%R^2^	95% CI	SV	%R^2^	95% CI		SV	%R^2^	95% CI		SV	%R^2^	95% CI	
Sex	0.005	3.37	(2.90, 4.90)	0.001	0.95	(0.75, 1.71)		0.001	0.71	(0.60, 1.09)		0.001	0.52	(0.49, 0.66)	
Education level	0.002	1.67	(1.36, 2.95)	0.011	7.73	(7.09, 9.97)		0.021	13.38	(12.78, 14.80)		0.019	11.15	(10.69, 12.43)	
Marital status	0.009	6.07	(5.39, 7.98)	0.034	23.79	(22.69, 26.76)		0.028	18.45	(17.80, 20.15)		0.007	3.91	(3.68, 4.66)	
Live alone (yes)	0.000	0.11	(0.06, 0.47)	0.005	3.41	(3.04, 4.58)		0.007	4.62	(4.34, 5.47)		0.004	2.40	(2.21, 2.93)	
Employment status	0.000	0.09	(0.08, 0.51)	0.003	2.21	(1.90, 3.39)		0.004	2.34	(2.14, 3.05)		0.002	1.11	(1.03, 1.39)	
Log household income	0.001	0.33	(0.19, 0.93)	0.001	0.79	(0.59, 1.40)		0.000	0.28	(0.21, 0.54)		0.000	0.13	(0.09, 0.27)	
Subjective health status	0.104	72.32	(70.47, 75.26)	0.068	47.06	(45.29, 50.42)		0.070	45.64	(44.70, 47.76)		0.096	57.25	(56.45, 59.25)	
Contact frequency with relative/family	0.007	4.70	(4.27, 6.61)	0.011	7.65	(7.08, 9.70)		0.010	6.19	(5.82, 7.30)		0.014	8.09	(7.74, 9.26)	
Contact frequency with neighbor	0.006	3.98	(3.63, 5.40)	0.005	3.59	(3.28, 4.98)		0.006	4.12	(3.86, 4.98)		0.007	4.44	(4.20, 5.31)	
Contact frequency with friend	0.011	7.35	(6.73, 9.80)	0.004	2.80	(2.51, 4.29)		0.007	4.28	(4.00, 5.33)		0.018	11.00	(10.59, 12.26)	
Total R^2^	0.144	100		0.144	100			0.154	100			0.167	100		

Abbreviations: SV, Shapley value; CI, confidence interval.

%R^2^ represents the relative importance value.

Aside from contact frequency, subjective good health was positively associated with happiness in all regression models and had the greatest relative importance value of all predictors across all age groups. The value of subjective health status was greatest at 72.32% in 19–29 years and lowest at 45.64% in 40–59 years. Happiness was negatively associated with log household income in all regression models. However, the relative importance value of household income peaked in 19–29 years and thereafter decreased with advancing age, whereas that of employment status increased from 19–29 years to 40–59 years.

## Discussion

Previous research emphasizes the role of social connections in happiness. Among Korean adults, satisfaction with family life was the most important predictor of subjective well-being [34]. Older Koreans, in particular, regarded being with family and good health as top two essential conditions for happiness in life [37]. To further examine the impact of various types of social contact on happiness, we specified regression models with contact frequency for various social relationships along with sociodemographic characteristics and subject health status as control variables, using publicly available nationwide survey data. Then, we applied the Shapley value decomposition method to measure the relative importance of different predictors of happiness by age group.

This present study shows that contact with the closest relative/family is more important to happiness than that with either the closest neighbor or the closest friend among middle-aged people aged 30–59 years old. However, contact with the closest friend becomes more important to happiness in young adults under 30 years and the 60 years and older group. This mirrors the finding that the relative importance value of the frequency of contact with the closest relative/family member increased from the 30–59 years to the 60 years and older group but only slightly, whereas the relative importance value of the frequency of contact with the closest friend has more than doubled from 30–59 years to 60+ years. Similar to the findings regarding the role of the closest family/relative is those of marital status. In our current analysis, marital status is a significant predictor of happiness in all age groups, which is consistent with the previous finding that the state of being married increases happiness [3]. However, the relative importance of marital status decreased by a considerable margin from the 30–59 years to the 60 years and older group. All these findings suggest that the roles of social contacts in happiness change in old age among adults after 30 years.

The changing relationship between social contact and happiness with increasing age can be supported by the earlier finding that family and friends have differential effects on well-being [38]. Doing activities with friends and family increases positive affect in old age. However, unlike family activities, doing activities with friends also decreases negative affect [38]. According to a study among Americans aged 65 years and older, contact with friends would lead to fewer discussions about stressful experiences than contact with family members [39]. With old age comes the loss of previous roles. The negative feelings associated with loss can be mitigated by frequent interactions with the closest friend.

While our findings support the critical role of friendship in happiness, it should also be recognized that there could be cultural differences in the relative importance of social contacts. Among African Americans, contact with neighbors appears to be the only significant predictor of happiness among all social relationships examined [14]. Similarly, the frequency of contact with children and friends was not a significant predictor of life satisfaction among Canadian adults [16]. Efforts have been made to explain the variations from a social policy standpoint. The positive relationship between the frequency of contact with family and relatives appears to be stronger in countries with greater public social expenditures on family benefits and services; Korea spends the least on family benefits and services out of 22 countries studied [18].

In addition to highlighting the role of social contacts, this present study reveals the changing impact of economic indicators on happiness. While financial security could be important to happiness in old age, happiness was negatively associated with household income and its relative importance diminished with advancing age. The latter finding is consistent with the findings of a previous study based on a number of other developed countries [1]. In comparison, another measure of financial security, employment status, was shown to be more important to happiness than household income in adults aged 30 years and older. These findings suggest that not just economic well-being but social participation associated with employment promote psychological well-being and healthy aging. On this note, there is evidence that working in old age lowers mortality [40]. Of course, it is possible that happiness is a cause of active employment rather than an outcome. Happy people reported having less sick days according to a randomized controlled trial, strongly suggesting that happiness makes people healthier [41]. One plausible explanation for the relationship between happiness and health is that happiness has a positive impact on health through playing a mediating role in the autonomic nervous system and promoting healthy behaviors [42].

In the literature, there is abundant evidence on the positive impact of social relationships on happiness. However, little has been known as to whether the type of social interactions contributes differently to happiness across age groups. Against this background, this study revealed that the frequency of contact with the closest friend is more important to happiness than that of contact with the closest relative/family in old age. These findings, however, should be interpreted with caution due to the following limitations. First, this study relied on cross-sectional data, which makes it problematic to draw causal inferences. Second, we used self-reported data, which may not accurately reflect the truth. Third, this study relied on a single measure of happiness and one dimension of social relationship, namely, contact frequency. This precluded a comprehensive examination of complex concepts of happiness and social relationship. Nonetheless, this study provides a rare glimpse into the changing relative importance of friendship to happiness as people age.

## Conclusion

This paper examined the influence of contact frequency on happiness and estimated the relative importance values of type of social contacts in happiness, using data from a nationally representative sample of Koreans and a variance decomposition technique. This study found that the frequency of interacting with the closest relative/family, neighbor, and friend is important to happiness across all groups. Moreover, this study highlights the increasing importance of friendship in happiness in old age. For aging individuals to maintain or increase their friendship activities would be beneficial to their psychological well-being. These findings may inform social policy intended to promote successful aging in an aged society.
